# White matter dementia then… and now

**DOI:** 10.3389/fneur.2022.1043583

**Published:** 2022-11-21

**Authors:** Christopher M. Filley

**Affiliations:** Behavioral Neurology Section, Department of Neurology and Psychiatry, University of Colorado School of Medicine, Marcus Institute for Brain Health, Aurora, CO, United States

**Keywords:** white matter, dementia, cortex, connectome, Alzheimer's disease, chronic traumatic encephalopathy, plasticity, brain health

## Abstract

White matter dementia (WMD) is a concept introduced in 1988 to highlight the importance of white matter pathology in producing cognitive dysfunction and dementia. Whereas gray matter, particularly the cerebral cortex, has been primarily investigated in the dementias, subcortical pathology has long been correlated with cognitive loss, and a corticocentric perspective cannot account for the full range of neurobehavioral disorders. Within the subcortical regions, white matter is prominent, accounting for about half the volume of the adult brain, and many white matter diseases, injuries, and intoxications can produce cognitive dysfunction so severe as to justify the term dementia. Recognition of this novel syndrome relied heavily on the introduction of magnetic resonance imaging (MRI) that permitted *in vivo* visualization of white matter lesions. Neuropsychological studies clarified the clinical presentation of WMD by identifying a profile dominated by cognitive slowing and executive dysfunction, and a precursor syndrome of mild cognitive dysfunction was proposed to identify early cognitive impairment that may later evolve to WMD. As knowledge advanced, the role of white matter in structural connectivity within distributed neural networks was elucidated. In addition, highlighting the frequent commingling of gray and white matter involvement, white matter pathology was associated with neurodegenerative diseases such as Alzheimer's disease and chronic traumatic encephalopathy, with potentially transformative clinical implications. In particular, preventive measures and treatments exploiting white matter restoration and plasticity are gaining much attention. Today, WMD has matured into a concept that not only integrates knowledge from across the spectrum of clinical neuroscience, but also informs new investigations into many perplexing disorders and enables a more complete understanding of brain-behavior relationships.

## Introduction

The idea of white matter dementia (WMD) was put forth in 1988 to call out the largely unrecognized importance of white matter pathology in producing cognitive dysfunction (1). At the time of this proposal, a distinction between cortical and subcortical dementia was popular in behavioral neurology (2), based on reports of dementia in progressive supranuclear palsy (PSP) (3) and Huntington's disease (HD) (4)—both of which feature major pathology in subcortical gray matter structures—that showed a deficit profile distinct from that of the classic cortical dementia Alzheimer's disease (AD) (5). In contrast to the amnesia, aphasia, apraxia, and agnosia of AD (5), the pattern in PSP and HD featured slowed cognition, forgetfulness (that would now be regarded as a retrieval deficit), personality changes, and what would now be termed executive dysfunction (3, 4). In this setting, relying primarily on a foundation of clinical, magnetic resonance imaging (MRI), and neuropathological observations in toluene leukoencephalopathy, my colleagues and I recognized that pathology confined to the cerebral white matter could also produce dementia (1). This concept thus led to the proposal of a new syndrome that departed from the conventional understanding of dementia, and our idea, form a different perspective than the powerful corticocentric bias in clinical neuroscience (6), met with only modest enthusiasm. As time and knowledge advanced, with MRI and neuropsychological testing regularly demonstrating correlations between white matter lesions and cognitive loss, the construct of WMD slowly attracted more attention (7–12), and this issue of *Frontiers in Neurology* embraces the concept with a series of articles dedicated to the topic. In this introductory paper, I will offer a focused review of the original formulation of WMD, its clinical characterization, its importance in the study of connectivity and distributed neural networks, its relevance to neurodegenerative diseases, and new perspectives in treatment, prevention, and recovery.

A foundational tenet of the WMD idea is that white matter and its disorders are most usefully studied by employing the scholarship of integration (13), so that the neuropathological origin of cognitive loss from any white matter disorder takes center stage in the quest to gain greater insight into how dementia in this context begins, and then evolves over time. White matter dementia is not a specific disorder diagnosable in clinical practice any more than is cortical or subcortical dementia, but rather a theoretical construct intended to expand thinking about the origin and progression of cognitive decline. By appreciating WMD as a recognizable syndrome, clinicians and investigators can reframe their approach to dementia, and pursue novel ideas on treatment and prevention that are not apparent from a corticocentric perspective (6). Moreover, in contrast to a reductionistic viewpoint in which one disease is studied intensively, WMD represents a synthetic perspective that highlights a brain region in which many disorders originate so that novel transdiagnostic insights can be revealed. Thus the scholarship of integration (13) lies at the heart of WMD, enabling consideration of a wide array of etiopathogenetic factors that help inform how the brain devolves from healthy cognition to a state of dementia. In its broadest sense, WMD contributes to the fundamental goal of behavioral neurology, which is how brain-behavior relationships are to be fully understood.

## Origin of the concept

The syndrome of dementia has been a recognized medical concept since antiquity (14), but the first use of the word in the modern sense was by the French psychiatrist Phillippe Pinel (1745–1826) in the eighteenth century (15). In terms of neuropathology, senile (neuritic) plaques and neurofibrillary tangles were famously recognized by Alois Alzheimer in 1907, and because of their location in the cerebral cortex (16), AD became widely known as a cortical dementia (5). Some years later, the term subcortical dementia was introduced to refer to a different dementia syndrome related to subcortical gray matter pathology in progressive supranuclear palsy, HD, and Parkinson's disease, among others (2–4). As the twentieth century progressed, the dichotomy between cortical and subcortical dementia became well established, and still finds use in neuropsychological assessment today. Interestingly, white matter diseases such as multiple sclerosis (MS) were also included in the subcortical dementias (2), albeit with less emphasis.

White matter as a distinct brain tissue was first recognized in 1543 by the gifted Italian anatomist Andreas Vesalius in the seventh book of his masterwork *De Humani Corporis Fabrica* (17). Here the white matter of the cerebrum is clearly demarcated from the more superficial cortical gray matter. Although recognized as a significant neuroanatomic entity—about half the volume of the adult brain—white matter was generally seen for centuries as a supportive tissue with no substantial role in the human behavioral repertoire. Some attention to white matter and behavior did appear in the nineteenth century, including seminal observations of Jean-Martin Charcot regarding cognitive and emotional alterations in MS (1), yet despite these developments, work on the dementias became strongly focused on the cerebral cortex as a result of the plaques and tangles found by Alzheimer in 1907 (16). In neuroscience broadly considered, cortical, and to a lesser extent subcortical, gray matter came to dominate the study of cognition and emotion (6).

The idea of WMD was conceived in this context, and naturally appeared foreign because it brought together two concepts that had not been formally conjoined. Adding to the problem of an unexpected association, the new idea began in the unusual clinical setting of substance abuse. Behavioral neurologists, while regularly engaged in the care of AD and traumatic brain injury, are not as often involved with individuals who abuse drugs. Remarkably, however, in evaluating patients with neurologic complications of solvent vapor abuse with the then novel technology of MRI, it became apparent that white matter damage in the brain was prominent and clinically significant. Therefore, the opportunity to examine individuals who presented with a unique dementia syndrome arising from toxin-induced white matter pathology—distinct from cortical dementia and the subcortical gray matter dementias—proved compelling.

Solvent vapor abuse is a recognized but under-appreciated form of drug abuse prevalent in persons of lower socioeconomic status. Fumes from spray paint or glue are inhaled for their euphorigenic effect, and often, in our patients, in prodigious quantities over many months or years. The favored substance was spray paint because of its low cost and ready availability, and our analysis of the inhaled fumes disclosed that the major solvent was toluene (18). This simple hydrocarbon, a highly lipophilic molecule that readily penetrates the brain, was shown to produce severe dementia in heavy abusers that was correlated with diffuse cerebral white matter involvement seen on MRI (19, 20). Supporting the MRI studies were autopsy data of affected individuals that documented selective white matter disease with sparing of both cortical and subcortical gray matter (21). This dramatic disorder—which we termed toluene leukoencephalopathy (TL) (22)—made it hard to escape the conclusion that pure white matter pathology can indeed cause dementia.

The discovery of TL prompted consideration of other neurologic disorders affecting white matter, and it was evident that dementia could also occur in these conditions (1). Although white matter-behavior relationships relevant to dementia are most evident in TL, the contribution of white matter pathology to dementia in MS, Binswanger's Disease, traumatic brain injury (TBI), the acquired immune deficiency syndrome, alcoholism, and normal pressure hydrocephalus all seemed plausible and worthy of further investigation (1). This first statement of WMD led to further investigation designed to detect more information about the relationship of white matter and behavior.

At that point, a transdiagnostic approach across all the white matter disorders was undertaken to seek clinical-pathological commonalities that might support a uniform and consistent role for white matter in the representation of cognition and emotion. This task presented a major challenge because WMD was a construct for which even basic data on incidence and prevalence were unavailable, a situation that continues today. Detailed literature review, however, disclosed that prominent or exclusive white matter pathology can be found in a diverse range of conditions including genetic, demyelinative, infectious, inflammatory, vascular, toxic, metabolic, traumatic, neoplastic, and hydrocephalic disorders (23, 24). The broad scope of neuropathological involvement is remarkable; the total number of white matter disorders numbers in the hundreds, and notably, some form of cognitive or emotional dysfunction can be found in all these disorders (23, 24). This observation is all the more impressive given that clinical reports of affected patients often focus on motor, sensory or other aspects of the disorder that are regarded as more clinically relevant, while neurobehavioral aspects are less emphasized (23, 24). Based on these combined observations, how it is that white matter pathology in general impacts cognitive function called out for further study.

## Neurobehavioral features

The next step was to examine whether WMD manifested with a distinct cognitive profile. Form the first encounters with TL patients, and with others who had white matter pathology (1), it was clear that prominent neurobehavioral features were slowed processing speed and executive dysfunction with relative sparing of language. Indeed, this profile was borne out in detailed neuropsychological studies of WMD disclosing a profile of cognitive slowing, executive dysfunction, sustained attention deficits, impaired memory retrieval, visuospatial dysfunction, and psychiatric disturbance, with relatively spared language, extrapyramidal function, and procedural memory (23, 24). Key studies establishing the singularity of WMD as a distinct syndrome were comparisons of MS with the classical cortical dementia of AD (25) and of MS with HD, a prototype subcortical dementia (26). Whereas MS differed from AD by its prominence of psychomotor slowing and inattention in contrast to episodic memory and language deficits (25), it differed from HD by the sparing of procedural memory (26). Thus WMD appeared to be a syndrome distinguishable from both cortical dementia, and from subcortical dementia arising from gray matter pathology.

As time went on, cognitive slowing and executive dysfunction emerged as the most prominent clinical features of WMD (23, 24). With respect to cognitive slowing, the normal physiologic function of myelin to increase axonal conduction velocity left little doubt that slowed processing speed would be expected to result from white matter dysfunction or damage, and indeed this prediction held up in later studies (27). Executive dysfunction also became established as a frequent consequence of white matter pathology, as the frontal lobes are extensively connected with other regions of the brain, and white matter involvement interferes with the organization of cognition that characterizes executive function (28). Another key feature of WMD is the relative preservation of language, which is generally the case albeit with some exceptions (23, 24). The sparing of procedural memory in WMD is noteworthy, although testing of this aspect of memory is a research method not routinely performed in the clinic.

The neuropathological basis of WMD merits comment. White matter disorders typically occur in a widespread distribution, with multiple cerebral regions in both hemispheres involved. Lesions are often multifocal, and may in more severe cases be confluent, and solitary white matter lesions are uncommon. While this lack of specific lesional predilection may suggest that no unifying clinical profile of WMD can be meaningful, no such criticism has been directed at the syndrome produced by the prototype cortical dementia of AD, which features widespread cortical pathology (5). Indeed, the similarity of clinical features across the spectrum of WMD disorders argues for a specific cognitive profile (23, 24). If amnesia, aphasia, apraxia, and agnosia can be accepted as signifying diffuse cortical pathology (5), cognitive slowing, executive dysfunction, relative sparing of language, and preserved procedural memory can surely be endorsed as evidence of diffuse white matter pathology (23, 24). An aspect of WMD warranting further study is the impact of variable lesion location and severity related to specific pathology, which is likely to introduce variability within the cognitive profile of affected patients.

At this point, however, an important caveat is that few cognitive disorders feature pure white or gray matter pathology. Indeed, most patients with cognitive dysfunction or dementia have a complex pattern of neuropathology that features some combination of white and gray matter involvement. This confound explains why many patients have overlapping cognitive deficits that blur the distinctions between various disorders. Whereas pure WMD does at times occur, it is likely uncommon, and among the most useful aspects of the concept is that it serves as a reminder not to neglect white matter pathology in the clinical picture.

## Mild cognitive dysfunction

From the first clinical experiences with disorders associated with WMD, it was apparent that many, if not most patients with white matter pathology on MRI do not have dementia, and instead, often have a milder syndrome that is symptomatic and measurable but not disabling. Thus white matter pathology was conceptualized as often involving a lesser degree of white matter hyperintensity on MRI, or even subtle white matter changes in the normal-appearing white matter (NAWM). First described in MS, the NAWM refers to subtle microscopic neuropathology not apparent on conventional MRI but still clinically significant (29), and further study found that NAWM abnormalities can be detected in many other diseases.

Accordingly, my colleagues and I turned to the model of systemic lupus erythematosus (SLE), an inflammatory disease in which white matter of the brain can be affected pathologically long before dementia develops. In patients with non-neuropsychiatric SLE (non-NPSLE), it was found using the technique of magnetic resonance spectroscopy (MRS) that myelin abnormalities—as indexed by elevated choline—in the frontal lobe NAWM correlated with impaired attention and working memory (30). We posited from these MRS data that early inflammatory myelinopathy was the source of cognitive decline in non-NPSLE that was sufficient to be detectable with cognitive testing but not severe enough to qualify as dementia. These observations led to the introduction of the term mild cognitive dysfunction (MCD) as a descriptor of early cognitive loss related to subtle white matter disease that could go on to dementia if treatment was unable to arrest the process (31). Although described in SLE, the concept of MCD could apply to any white matter disorder that is encountered at an early stage (31). This concept, which pertains to many diseases in which a high priority is to find early clinical manifestations that may have effective treatments, has the potential to stimulate further search for nascent white matter pathology in many disorders that would be much more readily treated while pathology is not yet advanced.

A good example of white matter pathology that may indicate an avenue for treatment is ischemic white matter disease, typically manifested by what is commonly termed white matter hyperintensities (WMH). These lesions are commonly seen on MRI scans of older people, and cognitive loss can develop when a sufficient burden of WMH accumulates (11). Recent studies have shown that subjective memory complaints in cognitively normal older people are associated with larger WMH volumes on MRI (32), suggesting that even before measurable deficits develop, white matter lesions could be producing symptoms of further cognitive decline in the future. The early treatment of WMH as a means of preventing WMD will be considered later in this review.

## Distributed neural networks and connectivity

In the years since WMD was proposed, the neuroscientific community has realized with ever greater conviction that the connectivity of the brain is a critical feature of its structure that helps determine its function. An early and highly influential expression of this notion was evident in the concept of distributed neural networks, large-scale assemblies of gray and white matter structures that were proposed to subserve cognitive domains such as attention, language, and memory (33). A similarly important conceptualization was that of frontal-subcortical networks (34). These networks are made up of assorted cortical and subcortical regions, all structurally connected so that the various regions operate in concert to enable the specific cognitive domain. In this context, the development of functional MRI (fMRI) attracted much attention because it could allow the localization of cortical regions involved in cognitive tasks (35). Functional MRI and related techniques are most useful for identifying cortical areas, and the pathways connecting them can only be inferred by examining the co-activation of regions between which white matter tracts are thought to make connections. Structural connectivity involving white matter clearly exists, however, and distributed neural networks doubtless include white as well as gray matter components. As time went on, the development of diffusion tensor imaging (DTI) allowed for the imaging of white matter tracts as a compliment to the gray matter imaging of fMRI (35). Two of the most clinically relevant examples of networks where white matter plays a central role are the left hemisphere language network, in which the arcuate fasciculus is important (36) and a right hemisphere social cognition network, in which the uncinate fasciculus has emerged as critical (37). Structural connectivity also holds true for recently described default mode, salience, and executive control networks (38).

The study of brain connectivity received a major boost in 2010 with the launch of the Human Connectome Project (HCP) by the United States National Institutes of Health (39). The HCP has been an ambitious collaborative effort to map out the entirety of connections in the human brain (39). This goal is addressed by the use of fMRI to establish functional connectivity between gray matter regions, and, particularly important for WMD, the use of DTI to map the structural connectivity provided by white matter (39). The HCP has been remarkably successful, generating an impressive, publicly available data and leading to over 1,500 papers (39). The imposing task of mapping the enormous complexity of white matter will not have immediate clinical benefits, but with time, it is not unreasonable to anticipate that the information generated by the HCP will be applicable in clinical settings where the goal is to identify individual tracts, the pathology they may harbor, and the clinical features that result.

An intriguing aspect of white matter connectivity is that it appears to be an evolutionary advantage particularly evident in the human brain. Comparative neuroanatomic studies have determined that cerebral white matter volume has actually increased over the course of evolution more than that of cortical gray matter (40, 41). Compared to other animals, the human brain thus has not only the advantage of more cortical neurons but also more connectivity between cortical regions. While brain size is one determinant of cognitive capacity, humans have smaller brains than large mammals such as whales and dolphins, and it seems likely that the most intelligent brains have the most cortical neurons and the greatest amount of white matter (42). Among many implications of this evolutionary feature is that humans may be particularly adapted for the operations of social cognition because extensive myelination enables the rapid behavioral response so critical for avoidance of predators, child-rearing, and—later in evolution—the nuances of interpersonal interaction (43, 44). That is, the high degree of cerebral myelination in *Homo sapiens* is necessary for the essential domain of social cognition, recently recognized as crucial for humans who must cooperate in complex social environments where rapid and accurate emotional perception and decision-making are of paramount importance (43, 44). Social cognition is lateralized to the right hemisphere (43, 44), where the right uncinate fasciculus plays a central role in linking gray matter structures of the social cognition network (37, 43, 44).

## Relevance to neurodegeneration

The disorders first associated with WMD all featured substantial neuropathology in the white matter, which provided a major distinction from the neurodegenerative diseases that are widely regarded as mainly involving the gray matter, most notably of the cerebral cortex (1, 5, 23, 24). However, recent findings have suggested that white matter pathology—recognized to a large extent because of advances in neuroimaging—may be fundamental in the etiopathogenesis of several neurodegenerative diseases. In these diseases, it is plausible that early white matter changes play a role in the initial stages of pathogenetic evolution, either preceding or accompanying gray matter pathology. This concept introduces a novel way of thinking about neurodegeneration, such that the gray matter changes seen late in the course, when patients typically come to clinical attention, may in fact be consequent to earlier pathologic alterations in white matter. Three examples of neurogenerative diseases that feature prominent white matter pathology are AD, chronic traumatic encephalopathy (CTE), and fragile X tremor-ataxia syndrome (FXTAS).

Research on AD, the most common cause of dementia in older people and a major health challenge around the world, is dominated by the amyloid cascade hypothesis, which posits that the deposition of insoluble amyloid β42 (Aβ) initiates a cascade of events that then include tau deposition, neurodegeneration, and dementia (45). Yet the repeated failure of drugs that reduce brain amyloid and tau to exert any meaningful effect on dementia has cast doubt on this notion (46). In particular, the reduction of brain amyloid appears to produce no substantial improvement in cognition, as shown by a meta-analysis of 14 randomized controlled trials (47). Work on the amyloid hypothesis continues, however, and monoclonal antibodies against amyloid may still find a place in AD treatment. One alternative proposal has been that white matter change may be the initial event in the pathogenesis of AD. In 2011, an influential paper of George Bartzokis presented the “myelin model of AD” in which early myelin pathology was posited to lead to the cortical pathology of neuritic plaques and neurofibrillary tangles (48). In the myelin model, which clearly remains preliminary, age-related loss of myelin prompts a homeostatic response in the brain that can eventuate in the development of AD pathology, with cortical protein deposition regarded as a later event that follows white matter involvement related to aging and associated vascular, traumatic and other injury (48). Degeneration of cerebral white matter has been observed at autopsy in the brains of normal individuals with AD pathology (49), and axonopathy and transport deficits have been found in brain regions with neither amyloid nor tau (50). MRI studies have found vascular white matter lesions in up to 90% of AD patients (48), and the total volume of these lesions has been directly associated with the odds of harboring AD neuropathology (51). An increase in MRI vascular lesion volume has been noted years before the onset of AD, or even of MCI (52), and these lesions have been shown to lead to overlying cortical atrophy in patterns typical of AD (53). With respect to white matter microstructure, DTI studies have demonstrated a decline in white matter integrity before the appearance of AD dementia (54), and diffusion kurtosis imaging (DKI) has also found greater diffusion restriction in preclinical AD (55). Another factor is age-related myelin loss, known as white matter retrogenesis, which is thought to produce a vulnerable substrate for acquired insults (56). According to the myelin model, early white matter injury superimposed on retrogenesis leads to AD through failed myelin repair mechanisms that produce cortical amyloid and tau as by-products (48). Indeed, animal experiments have shown that after induced stroke, the enzyme β-secretase reacts by activating neuregulin, a normal response protein important for myelin repair, but because β-secretase also cleaves Aβ from its parent molecule amyloid precursor protein, excess Aβ is deposited, followed by tau (57), consistent with the myelin model (48). With repeated injury, as can be expected in human aging, this overwhelming of myelin homeostasis leads to ongoing cortical damage (48) with Aβ and tau deposition producing toxicity that eventually leads to synapse and neuronal cell body loss in the hippocampus and neocortex, and the appearance of the amnesia, aphasia, apraxia, and agnosia of AD (5). Intriguingly, more recent imaging studies have shown that white matter changes, either increased WMH on MRI (58, 59) or lowered structural connectivity on DTI (60), are found in carriers of autosomal dominant AD genes who are cognitively normal, extending the possibility of early white matter involvement even to genetic AD. Thus in both sporadic and genetic AD, white matter pathology may be a core pathogenetic feature.

As provocative as this hypothesis may be, however, it should be interpreted in the context of many ideas being investigated on AD pathogenesis, including vascular, inflammatory, and other mechanisms that could implicate gray matter, white matter, or both (46). The etiology of AD is not well understood, and a broad perspective is needed to complement the amyloid hypothesis. In the white matter, the myelin hypothesis discussed above relies substantially on vascular disease as an early event in AD pathogenesis, and it is noteworthy that AD brains commonly harbor co-existent vascular pathology that often manifests in life as WMH (51–56). Alternatively, however, WMH in AD patients may reflect Wallerian degeneration subsequent to cortical pathology, or the impact of amyloid angiopathy on perivascular white matter (51, 56). Despite their variable origins, WMH have been found to increase the severity of dementia in AD (51). Another factor affecting white matter is genotype, as data have shown, for example, that the apolipoprotein E (APOE) protein functions as a cholesterol transport molecule that may help or hinder myelin repair depending on the allele present (48). White matter is a complex tissue under the influence of many environmental and genetic variables, and more study will clearly be needed to understand the complex relationships between white and gray matter pathology in this disease.

Another, and highly publicized disorder in which identifiable white matter pathology may initiate degenerative dementia is CTE (61). This is a condition in which frequent repetitive mild TBI in early life is proposed to produce a later dementia syndrome characterized by progressive tau deposition in the cerebral cortex. Most authorities concur that CTE closely resembles the older entity of dementia pugilistica in boxers, but the appearance of dementia and tauopathy in former American football players and military combatants has expanded the range of individuals who are considered at risk (62). As CTE is presently a neuropathological diagnosis, controversy exists about how it may be diagnosed in life, how common it may be, and who may be at risk (63). Despite these uncertainties, it is widely recognized that TBI of all severity is characterized by the white matter lesion known as diffuse axonal injury (DAI) (64–66), and DAI has been increasingly implicated as a trigger of post-traumatic neurodegeneration (66, 67). Recent DTI studies of mild TBI have documented microstructural changes in multiple white matter tracts that predict less favorable long-term outcome (68), and it is logical to posit that DAI precedes the tauopathy of CTE because tau is a normal constituent of microtubules within the axonal cytoskeleton (67, 69). The presumed origin of CTE in repetitive mild TBI thus strongly implies that injury to white matter is crucial in etiopathogenesis. Indeed, white matter pathology is present in all stages of CTE (61), and is considered likely, along with tauopathy, to contribute to dementia in CTE (70). While it is unclear which of these appears first in pathogenesis, one provocative report supported a white matter origin by documenting tauopathy in the frontal cortex overlying DAI in five schizophrenic patients who had undergone frontal leukotomy many years before (71). Thus DAI may lead directly to cortical tauopathy, and, by implication, attention to white matter injury early in CTE pathogenesis may be transformative. Even acknowledging the controversial status of CTE, a focus on white matter, and the DAI to which it is susceptible, may prove crucial in understanding the pathogenesis of this disease.

The last disease meriting discussion in this context is FXTAS (72, 73). This inherited neurodegenerative disease is caused by a trinucleotide (CGG) repeat expansion in the premutation range (55–200) of the fragile X mental retardation 1 (*FMR1*) gene, as opposed to the Fragile X syndrome (FXS), which is caused by >200 CGG repeats (72). While both FXTAS and FXS affect cognition, these diseases are clearly distinct. In addition to different age of onset and clinical manifestations, FXTAS and FXS have unique molecular pathogenetic features; FXS is related to transcriptional silencing with reduced or absent *FMR1* protein, whereas FXTAS is characterized by increased *FMR1* mRNA, which is thought to cause cellular injury *via* a toxic gain-of-function (72). In part because of the protective effect of the second X chromosome possessed by women, FXTAS is more common and severe in men (72). Cognitive dysfunction often occurs in conjunction with intention tremor, gait ataxia, and other clinical manifestations in FXTAS, and dementia can be disabling in many patients (72). Notably, FXTAS has been confirmed as an example of WMD in view of early executive dysfunction and slowed processing speed that are joined by memory retrieval deficit, impaired working memory, and psychiatric dysfunction with relative sparing of language (10). Autopsy of FXTAS patients has shown substantial white matter pathology (74), and involvement of the middle cerebellar peduncle (MCP) appears on conventional MRI as the “MCP” sign in about 60% of affected men and 10% of affected women (72, 73); the MCP sign has in fact become a major diagnostic criterion for the disease (73). Studies using DTI and MRS early in the course of FXTAS have found that changes in NAWM correlate with slowed cognition and executive dysfunction (75), indicating that white matter pathology may be an early developing event in pathogenesis (73, 75).

## Treatment

From a clinical viewpoint, treatment is of course a high priority, and a wide range of potential therapies exists to either reverse or improve the syndrome of WMD caused by its many specific etiologies. The broad spectrum of neuropathology that can impact the white matter naturally leads to the clinical dictum that the specific disorder causing WMD should be promptly treated with the best available therapies. The treatment of TL, with abstinence and supportive care, for example, differs from that of vitamin B_12_ deficiency, which responds to vitamin repletion. Reliance on conventional medical care for disorders causing WMD is covered in standard textbooks (76), and this vast topic is beyond our scope. Not all disorders leading to WMD have effective treatments, and patient care should be individualized once the diagnosis is made (76). One idea under study is use of a cholinesterase inhibitor such as donepezil, intended to augment ascending cholinergic transmission through white matter tracts that have been damaged (24, 66); however, whereas therapy such as this could be symptomatically helpful, it would not be expected to alter white matter structure.

In contrast, data are available to suggest that other treatments for disorders in all 10 categories of WMD (23, 24) can improve the structure of white matter, often with clinical improvement (77). Such treatments include hematopoietic stem cell treatment for metachromatic leukodystrophy, ocrelizumab for MS, highly active antiretroviral treatment for human immunodeficiency virus dementia, immunosuppressive drugs for systemic lupus erythematosus, abstinence for TL, vitamin B_12_ replacement, rehabilitation after TBI, steroids/radiation/chemotherapy for primary CNS lymphoma, shunting for hydrocephalus, and blood pressure control for leukoaraiosis (77). Thus therapy can be shown to improve damaged white matter regions and, in some cases, enhance cognition so that WMD can be averted or mitigated (77). These findings on the treatment of white matter lesions are preliminary, and more study is needed on the impact of treatment on cognition, but a recent study found that 40% of patients with substance abuse-related acute TL had improved MRI appearance, and partial or complete recovery over months to years, with abstinence and supportive care (78). Further experience with the potential for white matter pathology to respond to treatment will inform clinical practice and improve outcomes.

These observations stand in stark contrast to the common implication of dementia that it is irreversible. The lack of disease-modifying treatments in neurodegenerative dementias such as AD (46, 47) has unfortunately fostered a degree of therapeutic nihilism that overlooks the fact that dementia can in fact be reversible. Adopting the broad view that dementia is an acquired loss of cognitive and emotional abilities sufficient to interfere with daily functioning (5), it is clear that no implication of prognosis or reversibility need be presumed. Indeed, whereas AD and many related neurodegenerative disorders presently have no disease-modifying treatments, the treatment of WMD offers a different and more positive outlook.

A point to be emphasized in this discussion is that the treatment of white matter lesions is most efficacious when axonal structure is preserved (79, 80). The core constituents of a white matter tract are the axon and its surrounding myelin sheath, and when the myelin is damaged without axonal loss, the tract has the potential to remyelinate so that function can be restored. When the axons are damaged in addition to myelin, however, the clinical outcome is far less favorable (79). This observation was first made in MS (80), where the presence of MS “black holes” on brain MRI signifies axonal loss within demyelinative plaques, but the principle has widespread applicability to other white matter disorders as well (79).

## Preventive strategies

Prevention of dementia is of paramount importance given the absence of effective treatment for many affected people (46, 47). Dementia in this context primarily means AD, and it is recognized that most people with this disease also have vascular, Lewy body, and possibly other neuropathology (81). In previous years dementia was largely considered to be genetically determined, but the great majority of AD cases are still considered sporadic, and recent evidence has brought forth the possibility that some AD cases, particularly those arising later in life, may be prevented by attention to medical and lifestyle influences. Indeed, a recent review concluded that 40% of dementia may be prevented or delayed by attention to modifiable risk factors (81). As will be discussed, these factors all implicate white matter and the pathology to which it is vulnerable.

An important aspect of this topic is the notion of reserve, the capacity of the brain to withstand the onslaughts of multiple acquired insults so that brain health can be preserved (82). Classically divided into cognitive reserve—the life experiences that actively build resilience to brain insults—and brain reserve, the combined advantages of neuronal number, synapse density, and myelination that passively protect against pathology—the two are closely related, and often the single word reserve is used to refer to both. Importantly, evidence exists to suggest that salutary life experiences such as education, substantive work complexity, a social network, and leisure activities can override the genetic risk of AD conferred by the apolipoprotein E ε4 genotype (83). These benefits from life experiences again implicate the protection of white matter.

Evidence is mounting to show that white matter, like gray matter, is indeed a brain tissue for which reserve is an appropriate concept (84–87). Whereas acquired insults injure white matter and lead to cognitive loss, the avoidance of these insults not only helps avoid cognitive decline but also builds reserve for the subsequent mitigation of additional pathology. These considerations all suggest that white matter preservation is an increasingly desirable objective of preventive strategies (84–87). Indeed, protecting white matter from a wide range of acquired insults holds much promise (84–87). In light of the frequency with which white matter disorders appear at all ages, and the potential for reversibility of these disorders, there is growing enthusiasm for the idea that a public health approach is warranted with the goal of protecting white matter so that dementia can be averted (84–87). As discussed above, this approach may be feasible not only for dementia broadly considered (84–87), but for AD in particular (48).

It is now useful to consider the environmental hazards to white matter that compromise normal cognition and hence may lead to dementia. Evidence has been gathered to indicate that hypertension, diabetes, cigarette smoking, obesity, hyperlipidemia, unhealthy diet, physical inactivity, depression, sleep dysfunction, cognitive inactivity, social isolation, hearing loss, alcohol misuse, air pollution, and TBI are all associated with the accumulation of white matter disease (84–87). All of these problems have substantial potential for prevention by the gradual construction of reserve that implicates the maintenance of white matter health and integrity.

Given the public health dimensions of WMD and its prevention, an important observation about dementia prevention is that it seems to be effective. Epidemiological studies have produced evidence that the incidence and prevalence of dementia in some high-income countries, such as the United States, the United Kingdom, Sweden, France, and the Netherlands, have been declining in recent decades (88–90). While these trends are unfortunately not apparent in low- or middle income countries, the discrepancy may point to an explanation for the reduced burden of dementia in developed countries: these nations have improved education, health services, and social welfare that enhance brain health. From the perspective of WMD, the social advantages of living in a high-income country may be apparent through the health of white matter. The precise role played by these advantages in the protection of white matter is not entirely clear, but an interesting preliminary MRI study from the Netherlands showed that demographically similar older people had fewer white matter lesions and larger brain volumes in 2006 compared to 1995 (88). One specific intervention that has been examined in this regard is intensive control of systolic blood pressure—to less than or equal to 120 mmHg—which was shown in the SPRINT-MIND study to reduce the onset of mild cognitive impairment and slow the progression of WMH (91). These data imply that one factor contributing to declining incidence and prevalence of dementia may be white matter preservation made possible by the promotion of vascular health.

## Recovery of white matter pathology

As reviewed above, disorders causing WMD can at times be effectively treated with conventional therapeutics (77), and the relatively favorable treatment outcome in these cases appears to depend to a considerable extent on the preservation of axons within damaged areas of white matter (79, 80). With the axonal scaffolding in place, oligodendrocytes have the potential to remyelinate denuded axons, with a subsequent more favorable outcome (79, 80). More severe damage, in contrast, with loss of axons as well as myelin, indicates a worse outcome because the restoration of axonal projections is currently not possible. This capacity of white matter lesions to recover, as long as axonal structure is preserved, implies that early identification and treatment of disorders within the WMD spectrum is a high clinical priority.

Given the substantial potential for white matter pathology to recover, it is important to consider what interventions could be broadly applied to effect structural change. The perspective of this review precludes a complete account of novel treatments, but two major categories merit consideration. First is the restoration of vascular white matter lesions with conventional therapeutics, mentioned above (77) and potentially important in the global dementia epidemic (88–90), while the second is the use of emerging methods to remyelinate axons by exploiting the principle of plasticity, which could apply to people of all ages with white matter lesions.

An important recent development in the realm of WMD treatment has been the observation that WMH may regress over time, most notably with control of elevated blood pressure (92). Importantly, improved memory performance was noted in parallel with WMH regression over a 2-year period (92). These data add to the findings of the SPRINT-MIND study of hypertension treatment (91) by suggesting that treatment can not only prevent but reverse white matter disease while improving cognition concomitantly. Since the introduction of MRI in the 1980s, the appearance of presumably ischemic WMH in older peoples' brains has been widely recognized, and for many years these lesions were thought to progress and never improve, with consequent deleterious effects on cognition. Whereas WMH certainly can produce cognitive decline and dementia, regression of ischemic white matter lesions with medical treatment—and concomitant improvement in cognition—illustrates that attention to white matter may prove critical in averting the progression to dementia. The data supporting regression of WMH thus bolster the growing body of evidence that white matter is a promising target of treatment. White matter is increasingly seen as dynamic and adaptable under external conditions to which the brain is exposed, and improvement in vascular health is clearly salutary (88–90). As mentioned above, a key variable is likely to be the integrity of axons within WMH (79, 80); regression of WMH can be expected to be more robust with respect to small punctate lesions that feature slight axonal loss, as opposed to large confluent lesions in which extensive loss of axons is typical (93).

Another concept relevant to the treatment of WMD is the emerging notion of white matter plasticity. Plasticity, simply stated, is the capacity of an organism to change with experience, and in the brain, this idea is attracting much attention with the recognition that white matter is active and malleable tissue that can substantially remodel under the influence of external stimuli. While gray matter plasticity *via* synaptic remodeling, dendritic outgrowth, neurogenesis, and angiogenesis has been long appreciated (94), the propensity of white matter to manifest structural changes with environmental alterations has become abundantly clear from recent investigations. The core phenomenon underlying white matter plasticity is activity-dependent myelination, by which electrical activity in proximal axonal segments activates glutamatergic axo-oligodendroglial synapses to myelinate distal axonal segments (95, 96). Thus environmental experience can lead to more robust myelination and enhance network efficiency so that cognition may improve in parallel. This phenomenon has now been widely studied and can be observed in normal individuals at all ages and in several pathologic conditions (77, 97). Two instructive examples of white matter plasticity in normal people are bilingualism (98) and lifelong musicianship (99), both of which are associated with greater white matter integrity in multiple tracts bilaterally. With respect to pathologic states, white matter plasticity is only beginning to be explored in detail, but one noteworthy report showed that neurological music therapy in patients with moderate to severe TBI produced greater white matter integrity in several right hemisphere tracts and the corpus callosum with concomitant improvement in executive function (100). Activity-dependent myelination using physical therapy and cognitive tasks is also being explored in MS patients as potential means of remyelinative therapy (101), as long-sought pharmacological approaches to remyelination have remained elusive despite decades of effort (102). Also of interest in this context is the application of non-invasive brain stimulation with modalities including transcranial magnetic stimulation (TMS) and transcranial direct current stimulation (tDCS); whereas these methods mainly target cortical gray matter, and their impact on cognition is unclear, the possibility exists that these modalities may promote white matter plasticity and hence improve cognition (77).

Many questions require focused attention as the study of white matter therapeutics advances. Among the most important is the point in the natural history of the disorder at which treatment is most effective. The bulk of evidence suggests that early intervention is most likely to be efficacious, and so early diagnosis becomes critical. In this respect, WMD is no different from any other neurobehavioral syndrome.

## Future directions

With the construct of WMD in place, it is critical that investigators continue pursuing better understanding of all the specific disorders under this rubric with respect to their unique pathophysiology. We need to know not just that the WMD spectrum impairs neurobehavioral function in a predictable manner, but how the underlying neurobiology of each disorder within that spectrum can be better understood. Whereas neuropathology has greatly informed these diverse fields, *in vivo* studies of affected individuals will be crucial in explaining the neurobiological basis of each white matter lesion, whether it be macrostructural or evident in the NAWM. To accomplish these ends, the use of sophisticated neuroimaging including not only DTI, MRS, and DKI but also neurite orientation dispersion and density imaging (NODDI) (103), blood and CSF biomarkers such as the protein neurofilament light (NfL) that originates from the axons of myelinated fibers (104–106), and continued advances in the neuropathology of white matter disorders will be essential.

One question that has long persisted concerns the relationship of white matter lesions to gray matter pathology. In a progressive disease that involves both white and gray matter but has an uncertain pathologic sequence, which one comes first? Apart from the presence of white matter fascicles coursing within cortical and deep gray matter (107), lesions of which may lead to intrinsic gray matter injury, it is becoming increasingly evident that lesions within major tracts may lead to cortical pathology as a secondary event. Here a transdiagnostic approach may again prove helpful, as evidence from AD (53), CTE (71), and MS (108) can be collated to indicate that white matter lesions, by various mechanisms, may induce gray matter atrophy. A particularly useful example comes from MS, in which focal white matter tract lesions may cause both anterograde (Wallerian) degeneration toward the axonal terminal, or retrograde degeneration toward the cell body (109), producing gray matter damage in both directions and potentially widespread disruption of distributed neural networks. Advances in the understanding of these relationships may permit answers to important questions about the timing of white vs. gray matter pathology in diseases where the site of the initial pathology is still uncertain.

Another emerging area involves the genetics of white matter. Although acquired insults are attracting much attention, it is also clear that genetics plays a key role. White matter is under strong genetic control, with complex polygenic influences that impact the normal microstructure of axons, myelin, and oligodendrocytes, an individual's susceptibility to a variety of brain diseases, and responses to centrally-acting medications (110). In the clinic, it has been known since the 1990s that the apolipoprotein E ε4 (APOE_4_) allele is a risk factor for AD (111), and recent findings suggest that this variant may also increase the risk of CTE (112). Moreover, new evidence has suggested that a substantial component of WMH may be related to genetic factors (113), helping explain why adults with little or no vascular risk, and without migraine, may have notable WMH on MRI. In a development more relevant to younger individuals, genetic diagnosis is rapidly improving because of the emergence of exome sequencing for the more than 400 genetic disorders with white matter involvement (114).

With these advances will come more detailed insights into the etiopathogenetic origin and neurobehavioral sequelae of white matter damage, and exciting new therapeutic ideas. Among the most promising, but far from exclusive, avenues of study are vascular (28, 85, 115), traumatic (64–66), and inflammatory (54, 116) mechanisms, as these processes all impact white matter and can affect humans throughout the lifespan. As investigators pursue better understanding of these and other areas, clinicians will need to consider a host of related questions, including the making of an accurate diagnosis and determining what interventions are indicated, at what age, and at what stage of the disorder or its precursor stage. This combined information will not only assist in generating innovative treatments for highly prevalent disorders that can have a devastating impact on cognitive and emotional function, but also help us better understand white matter-behavior relationships.

Meanwhile, in addition to medical interventions (77), implementation of public health measures appears justified to foster white matter health (85–87). Such an initiative would apply not only to adults with ischemic WMH, but to people of all ages because of the ubiquity of TBI, air pollution, and other environmental hazards that are increasingly linked with white matter disease (85–87). The goal of optimal brain health—of which an important component is white matter integrity—can be sought not only by health care professionals working with their patients to offer appropriate medical care and encourage brain-healthy habits, but also, at a societal level, by policymakers and the public considering measures that can be widely applied to at-risk populations (85–87). The details of such an effort are complex and multi-faceted, involving a major effort aimed more at prevention than cure, but evidence is rapidly growing that adults can protect their brain with lifestyle interventions such as regular exercise, a healthy diet, and social activity (117). Moreover, in a welcome advance of social justice, recent data have suggested that a healthy lifestyle can reduce dementia risk in socioeconomically disadvantaged people (118), implying that this approach can extend beyond the developed world. Although most work on dementia prevention has focused on AD, encouraging data are beginning to appear on the benefits that modifiable risk factor reduction can produce in white matter (77, 87, 117). Thus prevention of white matter disease to avert the dementia that may follow becomes a legitimate social objective (87). More study of the impact of public health measures on white matter structure is needed to assess the potential for preventing dementia, but the substantial capacity for white matter tracts to recover with environmental interventions (77, 88–92, 95–97, 100) warrants an approach specifically intended to protect the myelinated systems of the brain.

## Conclusion

The conjoining of the clinical syndrome dementia and the neuroanatomic entity white matter in 1988, based on the constructs of cortical and subcortical dementia, was meant to expand knowledge and research on cognition by highlighting a sizeable portion of the brain that had not received sufficient attention (1). Even though Norman Geschwind, the founder of behavioral neurology, famously described in his classic 1965 paper how cerebral disconnection leads to neurobehavioral dysfunction (119), he considered neither the details of white matter neuroanatomy nor the neuroanatomic origin of dementia. The advent of MRI in the early 1980s was therefore central to the construct of WMD because of the possibility of detailed *in vivo* imaging of white matter and correlation of lesions with cognitive decline. Similar to the thinking implicit in the cortical and subcortical dementias, white matter was regarded as a tissue in which dementing disease can originate (1), and a brain region was thus seen as worthy of study as much as the individual disorders to which it is vulnerable (120, 121). Indeed, because a central principle of behavioral neurology is that similar symptoms and signs arise from affected brain regions regardless of the disease etiology (122), the synthetic investigation of all white matter disorders was aimed at disclosing new insights about the role of white matter in the pathogenesis of dementia broadly considered (1, 23, 24). In essence, by advancing the study of white matter-behavior relationships, WMD became a conceptually and clinically useful application of what has been termed the science of integration (13).

Despite a tenuous beginning, the idea of WMD has made much progress since 1988. The word dementia had been in medical use for more than two centuries (15), and white matter had been anatomically recognized for over 400 years (17), but the proposal linking these into one term was novel and unexpected (1). Yet the TL data were convincing (18–22), and together with later observations (23–28), it became undeniable that the approximately half of the brain comprised of white matter is essential for the higher functions of *Homo sapiens* (23, 24). Over time, the neuroscientific community increasingly adopted this perspective (7–12, 27, 48, 54–56, 87, 95, 96, 123). Although corticocentric myopia (6) persisted and likely hindered progress (124), a look back at the years since 1988 leads to the conclusion that the study of white matter and its contributions to behavior has substantially expanded. This perspective has even raised the arresting possibility that the neurobehavioral contributions of white matter may actually surpass those of gray matter, as suggested by a recent large study in which acquired focal lesions of densely connected white matter regions were more associated with impaired cognition than lesions of highly connected gray matter regions (125).

In retrospect, the WMD paper in 1988 (1) may be best viewed as reflecting nascent ideas on white matter and behavior that were being developed at the time, and that have advanced since then as behavioral neurology and related fields approached cognition and dementia with a broader perspective enabled by improved investigative techniques. Introduced at a time when myelinated tracts were paid scant attention in behavioral neurology, the idea of WMD served to emphasize the importance of white matter-behavior relationships in human cognition and emotion. The construct was never intended to minimize the importance of gray matter in behavioral neurology, and the co-existence of both gray and white matter pathology in most neurobehavioral disorders requires that a thoughtful, nuanced, and inclusive approach is best suited to advance our knowledge. In this crucial endeavor, white matter merits consideration as surely as gray matter in exploring human behavior and its disorders, and in the coming years further progress in this field can no doubt be anticipated.

## Author contributions

The author confirms being the sole contributor of this work and has approved it for publication.

## Funding

This work was supported by department funds from the University of Colorado.

## Conflict of interest

The author declares that the research was conducted in the absence of any commercial or financial relationships that could be construed as a potential conflict of interest.

## Publisher's note

All claims expressed in this article are solely those of the authors and do not necessarily represent those of their affiliated organizations, or those of the publisher, the editors and the reviewers. Any product that may be evaluated in this article, or claim that may be made by its manufacturer, is not guaranteed or endorsed by the publisher.
